# Long-Lasting Symptomatic Cerebral Hyperperfusion Syndrome following Superficial Temporal Artery-Middle Cerebral Artery Bypass in a Patient with Stenosis of Middle Cerebral Artery

**DOI:** 10.1155/2018/4717256

**Published:** 2018-09-23

**Authors:** Shinji Shimato, Toshihisa Nishizawa, Takashi Yamanouchi, Takashi Mamiya, Kojiro Ishikawa, Kyozo Kato

**Affiliations:** Department of Neurosurgery, Kariya Toyota General Hospital, 5-15 Sumiyoshi-cho, Kariya City, Aichi 448-8505, Japan

## Abstract

Cerebral hyperperfusion syndrome (CHPS) is a complication that can occur after cerebral revascularization surgeries such as superficial temporal artery- (STA-) middle cerebral artery (MCA) anastomosis, and it can lead to neurological deteriorations. CHPS is usually temporary and disappears within two weeks. The authors present a case in which speech disturbance due to CHPS lasted unexpectedly long and three months was taken for full recovery. A 40-year-old woman, with a history of medication of quetiapine, dopamine 2 receptor antagonist as an antipsychotics for depression, underwent STA-MCA anastomosis for symptomatic left MCA stenosis. On the second day after surgery, the patient exhibited mild speech disturbance which deteriorated into complete motor aphasia and persisted for one month. SPECT showed the increase of cerebral blood flow (CBF) in left cerebrum, verifying the diagnosis of CHPS. Although CBF increase disappeared one month after surgery, speech disturbance continued for additionally two months with a slow improvement. This case represents a rare clinical course of CHPS. The presumable mechanisms of the prolongation of CHPS are discussed, and the medication of quetiapine might be one possible cause by its effect on cerebral vessels as dopamine 2 receptor antagonist, posing the caution against antipsychotics in cerebrovascular surgeries.

## 1. Introduction

Superficial temporal artery (STA) to middle cerebral artery (MCA) anastomosis is a surgical procedure of direct revascularization to improve cerebral blood flow and potentially prevent brain infarction for patients with stenoocclusive cerebrovascular diseases [1]. While this surgical procedure has the advantage of rapid improvement of impaired cerebral blood flow (CBF), there is a potential risk for postoperative cerebral hyperperfusion syndrome (CHPS) [2, 3].

Cerebral hyperperfusion state is defined as a major increase cerebral blood flow (CBF) following surgical repair that is well above the metabolic demands of the brain tissue, and it can be detected using single-photon emission computed tomography (SPECT) [4]. When hyperperfusion reaches symptomatic state, CHPS is characterized by unilateral headache, facial, and ocular pain, seizures, and focal neurological signs [4, 5]. With regard to the time course of CHPS, the symptoms usually start during the acute stage after bypass surgery [6–8], and the duration is 1-2 weeks in most cases [6, 8, 9].

We report a case of a patient who suffered from speech disturbance for more than three months due to CHPS after the surgery of STA-MCA anastomosis for symptomatic left MCA stenosis. The characteristics of this case and the mechanism of the long duration of CHPS are discussed.

## 2. Case Report

**Figure 1:**
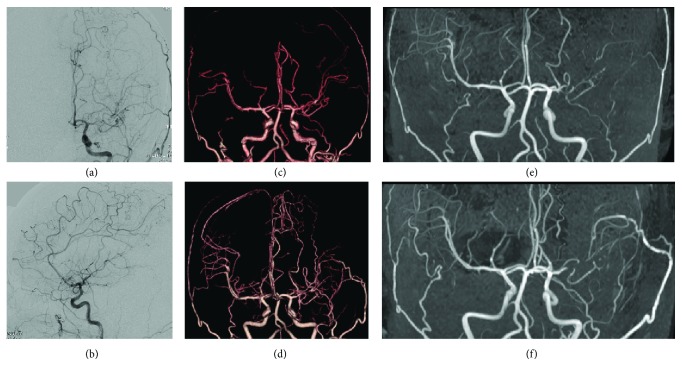
Pre- and postoperative cerebrovascular examinations. (a) and (b) Preoperative digital subtraction angiography showing narrowing in the M1 portion of MCA. (c) Preoperative 3D-CT angiography showing narrowing in the M1 portion of MCA. (d) Postoperative 3D-CT angiography showing the patency of STA-MCA anastomosis. (e) Preoperative magnetic resonance angiography (MRA) showing stenosis of M1 portion of left middle cerebral artery (MCA) and poor visualization of peripheral MCA. (f) Postoperative MRA showing the patency of STA-MCA anastomosis and improvement of blood flow in peripheral MCA.

A 39-year-old woman, who had been taking medication of quetiapine as an antipsychotics for depression, experienced mild dysarthria and visited the department of neurology in our hospital. Her symptom was diagnosed as drug-induced lip dyskinesia, which disappeared in a week. Screening head magnetic resonance imaging (MRI) at this time revealed stenosis of the left MCA with no brain parenchymal lesions (Figure 1(e)), why she was consulted to our department. We performed angiography, confirming moderate M1 portion stenosis (Figures 1(a) and 1(b)). SPECT showed no apparent laterality in CBF, thereby we decided to observe her with no treatment.

**Figure 2:**
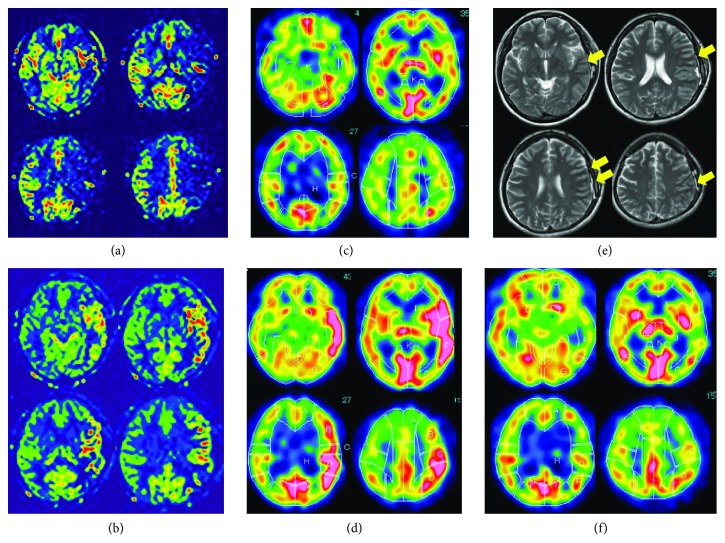
Pre- and postoperative neuroradiologic examinations. (a) Arterial spin labelling (ASL) of MRI showing decrease of CBF in the left brain when the patient presented with right hand weakness and numbness; (b) ASL on postoperative day (POD) 5 showing an increase of CBF in the left cerebrum; (c) preoperative single-photon emission computed tomography (SPECT) showing no apparent laterality in CBF. (d) SPECT on POD12 showing remarkable increase of CBF in the left cerebrum; (e) MRI T2WI on POD16 showing slightly hypointense change in the subcortex of the left cerebrum (arrows). (f) SPECT on POD33 showing normalization of CBF.

Seven months later, the patient experienced mild weakness and numbness in her right hand and visited our department. Although MRI showed no apparent ischemic change in her brain, arterial spin labelling (ASL) of MRI detected the decrease of CBF in the left cerebrum (Figure 2(a)), which was thought to well correspond for her symptoms. She was admitted and treated with an antiplatelet agent. Two weeks later, she still complained of numbness in her right hand; thereby, we decided to perform left STA-MCA anastomosis to prevent deterioration of her symptoms. Preoperative SPECT showed no apparent laterality in CBF (Figure 2(c)). On operation, left temporal craniotomy was performed, and the parietal branch of the STA was anastomosed with the M4 portion on the temporal lobe (Figures 1(d) and 1(f)). The intraoperative course was uneventful, and the patient recovered from anesthesia without any new neurological symptoms

Postoperatively, her speech was normal until postoperative day 1 (POD1). On POD2, she exhibited mild speech disturbance, which worsened day by day finally resulting in complete motor aphasia on POD6. Her comprehension was kept normal. On POD3, generalized convulsion occurred, which ceased quickly by diazepam, and levetiracetam was initiated. On the same day, she presented with mild weakness of right upper extremity, which improved gradually and disappeared on POD7. MRI and CT showed no ischemic or hemorrhagic changes, but ASL and SPECT revealed remarkable increase of CBF in the left cerebrum (Figures 2(b) and 2(d)), by which the symptoms were diagnosed as CHPS. Despite the treatment with strict blood pressure and the administration of edaravone and minocycline, complete motor aphasia remained unchanged on POD21. MRI showed no abnormality except slightly hypointense changes on T2 weighted images and FLAIR (Figure 2(e)). At this point, the patient was discharged partly because of the request from the patient, and we continued to follow her in outpatient visit. One month after the surgery, the patient started to utter words that were not fluent, when SPECT and MRI showed normalization (Figure 2(f)). Thereafter, the improvement of her speech was slow, and totally more than three months was taken for full recovery after the surgery.

## 3. Discussion

Cerebral hyperperfusion syndrome (CHPS) following cerebral revascularization is well recognized particularly in the context of carotid endarterectomy (CEA) and carotid artery stenting (CAS) [4, 10]. STA-MCA anastomosis, which can be indicated for atherosclerotic diseases as well as moyamoya disease [1, 11], is another surgical procedure in which CHPS can occur [2, 3]. The incidence of postoperative CHPS varies according to the surgical procedures or background of the patients, and it has been reported to occur in up to 50% of patients after STA-MCA anastomosis [2, 3, 10]. Resultant neurological deficits may be permanent and severe if intracranial hemorrhage occurs due to CHPS [12]. Otherwise, hyperperfusion is usually a temporary status that disappears within one week after surgery, and the symptoms are expected to disappear within 1-2 weeks in most cases [6, 8, 9].

In the present case, the initial presentation of CHPS appears typical in terms of the symptoms as well as the pattern of hyperperfusion on SPECT, because the area of hyperperfusion is frequently in localized brain cortex after STA-MCA anastomosis [6–8], and the focal neurologic signs in accordance with the anatomical location of the site of anastomosis are the most frequent symptoms in STA-MCA bypass surgery [6]. Then, hyperperfusion state detected on ASL or SPECT as well as complete motor aphasia continued for more than three weeks, which was longer than usually seen. More unexpectedly, the improvement of speech was slow even after the normalization of CBF on SPECT, requiring additionally more than two months for full recovery. The cytogenic edema could be a possible cause for persisting neurological deficits in patients with severe hyperperfusion [13]. In our case, although slightly hypointense change on MRI-T2 and FLAIR images was observed in the subcortex area of the left cerebrum, no changes indicating edema or stroke were seen at all. Epilepsy was denied as well by the fact that no seizures occurred after the initiation of levetiracetam and she was alert all the time. Therefore, we assume that her prolonged symptoms were the sequela of CHPS, and the full recovery of her symptoms could support it.

There are two cases showing long duration of CHPS after STA-MCA bypass reported in the literature, both of which were the patients with moyamoya disease [6, 14]. In one case by Takemoto et al., a 59-year-old female patient experienced 5 weeks duration of aphasia and right hemiparesis [14], and the period of hyperperfusion detected on SPECT was three weeks, which is similar to that in our case. In another case shown in the study of 27 consecutive patients by Fujimura et al. [6], a 37-year-old male suffered from 30-day duration of aphasia and sensory disturbance. In their case, SPECT was taken only on POD2 and 7, and the actual duration of hyperperfusion on SPECT is unknown. The total duration of symptoms due to CHPS in our case is the longest in the literature, and this is the first case in atherosclerotic diseases, to our knowledge.

Although underlying mechanisms for hyperperfusion remain undetermined, possible pathophysiology for CHPS has been suggested to be the impaired autoregulation, endothelial dysfunction mediated by free radicals, breakdown of the baroreceptor reflex, and breakdown of blood-brain-barrier (BBB), which results from a rapid increase in cerebral blood flow [15, 16]. The impaired autoregulation is thought to be the central mechanism because the reduction of preoperative CBF and cerebral vascular reserve capacity (CVRC), which lead to impaired autoregulation, are known as the significant risk factors for hyperperfusion [13, 15]. In the case reported by Takemoto et al. mentioned above, regional differences in the functional recovery of cerebrovascular reactivity were suggested to the possible cause, based on the observation that the area of hyperperfusion shifted from basal ganglia to the cortex surrounding the anastomosis for three weeks [14]. In our case, speech disturbance persisted long while paresis of right upper extremity disappeared on POD7 although relatively large area covering motor area for right upper extremity was affected. Subtle differences between regions in the functional recovery of cerebrovascular reactivity might have led to such discrepancy in the length of the symptoms, which image studies could not detect. Preoperative measurement of CBF with acetazolamide in addition to that at the resting state is demonstrated to be helpful to predict the development of CHPS [17], and it might have shown some evidences revealing impaired autoregulation with regional differences.

Positron emission tomography (PET) is another tool to detect hyperperfusion in different aspects than MRI or SPECT can detect, and the decrease of oxygen extraction fraction (OEF) and the tendency of high cerebral blood volume (CBV) are reported to be the factors observed on PET in patients who developed CHPS [18]. In particular, prolonged recovery of CBV values is suggested to have a key role in the development of hyperperfusion and the associated clinical symptoms [18]. Therefore, in our case, impaired oxygen metabolism or increased CBV might have persisted long even after the normalization of CBF.

Considering that the symptoms persisted extremely long despite all possible treatments including strict control of blood pressure and the administration of edaravone and minocycline [19–21], there might have been some particular factors in this patient. Here, in view of the daily medication of quetiapine that the patient had been taking for depression, we suggest one hypothesis that quetiapine, which has an antagonistic action on dopamine 2 receptor, might have affected cerebral vessels and prolonged hyperperfusion, based on the previous reports regarding the effect of dopamine 2 receptor antagonist (D2 antagonist) on cerebral vessels [22–24]. Ion et al. reported in a postmortem study for Schizophrenia patients that D2 antagonist may affect the neurovascular unit and small structural microvascular changes [22]. Another study with adult rats by Gepdiremen et al. showed that wall thickness of basilar artery decreased significantly due to vasoconstriction when haloperidol, D2 antagonist, was given [23]. In addition, similar antipsychotics are reported to be likely to incur cytotoxic effects and apoptosis of BBB endothelia with an impairment of barrier functionality [24]. In our case, the cerebral vessels had presumably been exposed to D2 antagonist for a long time; thus, they might have sustained the impairment of vasoconstriction or BBB, leading to prolonged dilatation of the vessels or impaired oxygen metabolism following the increase of CBF after STA-MCA bypass surgery. In this regard, we should mention that we restarted the daily medication of quetiapine immediately after the surgery and continued it throughout the course. The hypothesis mentioned above could not be verified, and no cases with any brain diseases have been reported in which antipsychotics is suggested to affect cerebral blood flow, to our knowledge. Therefore, the degree of the influence of quetiapine on CHPS in this case could not be extrapolated. However, our case with such rare relationship could pose the alarm regarding the management of antipsychotics for cerebral revascularization surgeries.

This case represented a rare clinical course of CHPS. Factors causing long duration could not be determined, but we suggest a possibility of the involvement of antipsychotics by its effect on cerebral vessels as D2 antagonist. Further studies are needed to elucidate the mechanisms of CHPS and to establish the optimal treatment strategy for better prognosis.
